# Understanding Chinese Gay Men’s and Lesbians’ Experiences of Coping with the Pressure to Marry from the Lens of *Suzhi* Discourse

**DOI:** 10.3390/ijerph19020796

**Published:** 2022-01-12

**Authors:** Xuan Ning, Sijia Guo

**Affiliations:** 1 General Education Office, Hong Kong Baptist University-Beijing Normal University United International College, Zhuhai 519087, China; xuanning@uic.edu.cn; 2 College of Public Administration and Humanities, Dalian Maritime University, Dalian 116026, China

**Keywords:** China, homosexuality, marriage, *suzhi*

## Abstract

Chinese gay men and lesbians are faced with multiple challenges by living in a heteronormative society, and marriage is considered to be a major concern among this group of people. Anchoring from the lens of *suzhi* discourse, this research carried out a qualitative study by interviewing 21 gay men and lesbians to explore how they refer to the *suzhi* discourse to justify their choices when faced with the pressure to marry, so as to align themselves more with the mainstream social values. Results indicate that when faced with the pressure to marry, gay men and lesbians refer to the *suzhi* discourse to construct a positive same-sex identity and civilized community, pursue civilized same-sex relationships, or construct a flexible life.

## 1. Introduction

Chinese gay men and lesbians are faced with many challenges posed by the heteronormative society they live in, among which, marriage is the primary concern for most of them [1]. As same-sex marriage is not legalized in China, the concern here refers to gay men and lesbians’ pressure to be in an opposite-sex marriage. Different from the situation in the West where the marriage discourse is associated more with personal choice, the heteronormative discourse in China has cultivated marriage as a mandatory obligation, which resulted from the interplay of symbolic significance and certain compulsory institutional constraints [2]. Dire consequences such as estrangement and suicide arise from the severe pressure to marry faced by gay men and lesbians, which deteriorates their social well-being [3]. A discourse of civilization has sprung up in recent decades, which cultivates individuals’ new consciousness based on civility and quality by emphasizing modernity, urbanity, wealth, knowledge etc. [4]. It plays an essential role in the processes of citizenship in contemporary China, facilitating individuals’ understanding of obligations, responsibilities, and rights, determining who are included in this set of rights and responsibilities and who are excluded [5]. *Suzhi* is a keyword of this discourse that is roughly translated as “quality” in English, indicating the nurtured and innate intellectual, moral, physical, and psychological qualities of people’s conduct and their bodies [5]. The term was conjoined with the idea of population in 1976 as the beginning of the economic reforms. It represents that the state policy shifted its focus from regulating the increase in the population to enhancing the quality of the population. It was a general explanation for China’s backwardness and potent call for development during this period. By 1990s, *suzhi* was enlarged to encompass social distinctions and articulate social boundaries. A person’s quality could be distinguished from his fellows by his practices of consumption and his social mobility to upper social class [6]. Affected by this discourse, Chinese gays and lesbians have formed new practices to adapt to this discourse of civilization. Existing research has rarely studied the civilization discourse in the Chinese *tongzhi* community, and the operation of *suzhi* among gays and lesbians in terms of dealing with the pressure to marry is understudied. In order to address these gaps, this research collected qualitative data from gays and lesbians to analyze how they deal with the pressure to marry by adjusting their practices to adapt to the discourse of civilization.

## 2. Literature Review

Studies on how Chinese gay men and lesbians resist the power influence exerted by the marriage institution are few, given how important this issue is. Research within this area mainly investigates reasons for gay men and lesbians to get married, how they deal with the marital pressure, and how their coping strategies influence people involved.

First, in terms of reasons to get married, social stigma resulted from being gay, single, and unmarried is the primary source of pressure that motivates gay men and lesbians to get heterosexually married [7]. Extant research documents that social stigma against sexually transmitted infections (STIs), especially AIDS/HIV, tilts people’s attitude towards homosexuality negatively [8]. Neilands et al. [9] engaged in a study on 477 men who had sex with men in Shanghai, and the results showed that the higher their self-stigma, the bigger chance they would marry heterosexual spouses. Traditional Chinese culture accentuates children’s showing filial piety to their parents, and the most critical way to realize this is through marriage and reproduction [10,11]. Therefore, this reason is listed as the primary one for most Chinese gay men and lesbians to get married [12]. In addition to the cultural values advocated by society, the marriage institution in many countries is closely associated with social welfare, rights, and duties; only by getting married could individuals attain their rights and execute their obligations that account for their sense of well-being. This heterosexist opportunity structure strictly promotes heterosexual relationships but discourages same-sex relationships [13].

For the coping strategies to alleviate their marital pressure, many Chinese gay men and lesbians choose to conceal their same-sex identity and marry heterosexuals to manifest their commitment to reproduction and filial piety [3,14]. Another emerging and under-addressed strategy is nominal marriage. This is a type of marriage in which a gay and a lesbian pretend to be a couple, primarily for familial, social, and reproductive purposes. Gay men and lesbians who are engaged in this marriage usually stay in a collaborative relationship [3,15,16]. However, unavoidable cases like family conflicts, domestic violence, and sexual abuse frequently happen under this circumstance [16]. In addition, some gay men and lesbians take an extreme approach by threatening parents with their suicidal ideation, and some do commit suicide. Further, becoming estranged from their families is also a common choice among this group of people in China [17,18].

## 3. Theoretical Framework

This study draws on ideas from the *suzhi* discourse that is widely prevalent in Chinese society. It contains and also interacts with other powerful key words like modernity and civilization. *Suzhi* is not only a normative goal or substance that is supposed to be attained by each individual; moreover, it is a value coding differentiating and highlighting gaps between rich and poor, good and bad, or civilized and uncivilized. The code often functions ideologically through antithesis. For instance, in terms of middle-class nurture, a single child is in sharp contrast with the massive migrant workers and social disorders they were raised with. Hence, parental investment is vital to distinguish their child from the derogated migrants. The quality of one’s child is what ensures an urban middle class family to not fall back to China’s hinterlands [6]. That is how *suzhi* is intertwined with China’s modernity and civilization. It plays an essential role in the processes of citizenship in contemporary society and contributes to people’s understanding of their claims, obligations, responsibilities, and rights which associate them with being members of a certain country. Moreover, it will produce discourses on what are and how we should treat “ideal” citizens and vice versa [19].

The marriage institution is a dominant discourse that builds on a heteronormative structure formed by legal and policy discourses that define heterosexual marriage as the only possible way to achieve certain rights, welfare, opportunities, and obligations. Hence, unequal power relations are inherent in these discourses with heterosexuals wielding power and occupying the hegemonic position, whereas gay men and lesbians are excluded for otherness. However, gay men and lesbians position themselves in this dominant discourse by generating their subjectified identities pressed by the dominant discourse. They formulate counter arguments against the hegemonic discourse and engage in power struggles with the so-called privileged and powerful actors to result in different kinds of negotiations with the powerful and privileged. *Suzhi* is such a taken-for-granted ideological discourse that it continues to operate even in marginalized communities. Individuals intend to normalize themselves by othering different communities. It is a hegemonic rhetoric which divides people into various social categories even within Chinese same-sex community. Within this marginalized community, *suzhi* can exercise its power to exclude the marginalized individuals, which points to an anxiety of improving its low-*suzhi* members [20]. One aspect that *suzhi* specifically emphasizes in contemporary China is people’s quality of morality, hence individuals’ proper expressions of their same-sex identity and practices are important to differentiate them from others who are low-*suzhi*.

China’s marriage institution rests on the heteronormative discourse assigns homosexuality negative meanings from the following dimensions. Heteronormativity is a discourse that advocates and promotes heterosexuality, gender conventionality, as well as family traditionalism as the appropriate and right way for social members to be [21]. Culturally, marriage associates itself with traditional cultural values of life-culmination attaining, familism, and filial piety. Therefore, unmarried people are regarded as unsuccessful, unfilial, and socially ostracized [22]. Institutionally, the state government’s policies since 1949 have created a hard and unfavorable living condition for gay men and lesbians. During the initial period of the new country’s construction process, the state government deliberately upheld altruism, which encouraged both men and women to work hard all through the day without being distracted or perplexed by private happiness [23]. Moreover, the state government tended to associate sexuality more with politics after 1949. Inside China, the ruling Communist Party denied the existence of homosexuality in China in order to maintain social stability. At that time, homosexuality tended to count as a decadent way of life that stemmed from capitalism, and anything related to capitalism was the enemy of socialism and thus should be condemned [10]. The state government proclaimed that homosexuality was an issue that only existed in the West [22]. At that time, anyone caught engaging in same-sex behavior would face horrible punishment including charges and administrative penalties and being persecuted and harassed by the police [19]. Nowadays, situations of Chinese gay men and lesbians have improved compared with the previous circumstances. Information on same-sex movements or the development of legal reform regarding the issue of homosexuality is beginning to come into China. Nevertheless, the state-run media still describe homosexuality as abnormal and perverted. When it comes to sensitive information like homosexuality, official channels in China would block it and release to people what they want people to know [24]. Right now, the Ministry of Culture and the State Administration of Radio, Film and Television stop the broadcasting of depictions of homosexual behaviors by imposing scrutiny [25]. As for the medical and legal dimensions, though homosexuality has been decriminalized and depathologized in 1997 and 2001, respectively, gay men and lesbians are still ascribed with the stigmatizing connotations of HIV-infected and mentally ill [25]. These negative connotations render gay men and lesbians a disadvantaged position in China by depriving them of certain rights and equal opportunities. In order to shield stigma and gain their citizenship, gay men and lesbians have to cultivate their own *suzhi* to be included in Chinese society and regarded as ideal citizens.

## 4. Method

The fieldwork was carried out in Guangdong, China, from March to June 2017. Data show that the Chinese gay population tends to reside in first-tier cities, especially in the southern part of China [26]. Besides, the world’s first and largest organization for LGBT people and their families PFLAG (parents, families and friends for lesbians and gays) founded its chapter in Guangzhou in 2008, which then became one of the most influential Chinese LGBT NGO. Due to the above reasons, Guangdong province with first-tier cities like Guangzhou and Shenzhen is a suitable province to conduct data collection. Data were collected through semistructured interviews with each lasting around one hour, with audiotaping and transcription. A Chinese coauthor who self-identified as heterosexual, fluent in English and Mandarin, and had built a rapport with several Mainland lesbian and gay community activists interviewed the participants in Mandarin—the participants’ first language. Participants were recruited through snowball sampling. The LGBT activists invited the interviewer to post recruitment information on the WeChat platform (a communication mobile app widely used among the Chinese population) where groups of gay men and lesbians socialize. The recruitment poster contained the researcher’s information, purpose of this research, types of interview questions, length of the interview, and payment of 50 RMB as honorarium for each individual’s participation. Altogether 21 gay men and lesbians participated in this study, whose ages ranged from 23 to 39 years, including 12 people who self-identified as gay men and 9 people who self-identified as lesbian. As this research aims to obtain as diverse opinions as possible, gay men and lesbians with different marital status and various relationship experiences were included for the purpose of generating a comparatively full picture. The study secured ethical approval by the Human Subjects of Ethics Subcommittee at the City University of Hong Kong, Hong Kong. All participants were informed of the aims and processes of this study and written consent was obtained. Participants’ confidentiality, anonymously coding their names for reporting purposes, and the choice of immediate withdrawal were guaranteed.

Among the gay participants, two had the experience of nominal marriage, three had a same-sex partner, and five were single. For lesbian participants, one had marital experience, three had a same-sex partner, and four were single. The participants’ occupations varied from civil servants to college students and white-collar workers to CEOs.

Each respondent was assigned a number: for gays, G1, G2, etc.; for lesbians, L1, L2, etc. Questions seeking their opinions, attitudes, and actions—such as “What kind of marital pressure have you encountered?” “How do you cope with your marital pressure?” “How do you view homosexuality?” “How do you perceive marriage?” “Why do you think and behave in such a way?”—were asked to invite participants to share answers regarding power and resistance.

The interview data were managed and analyzed by NVivo. The researcher who did the interviews coded all the transcripts. Data analysis initially focused on the influence of civilization discourse and participants’ social practices in terms of dealing with the marital pressure. Once key codes were constructed, sub-codes and categories were further examined and developed. Direct quotes will be used to support the authors’ assertions.

## 5. Findings

The data analysis discovered how Chinese gay and lesbian participants referred to the *suzhi* discourse to make themselves more in aligned with the mainstream social values so to make themselves more acceptable to Chinese society. To show their highly cultivated *suzhi*, participants took three approaches—constructing a positive same-sex identity and civilized community, pursuing civilized same-sex relationship, and constructing a flexible life.

### 5.1. Constructing a Positive Same-Sex Identity and Civilized Community

In order to make their ideology become a taken-for-granted piece of knowledge, participants interpreted homosexuality with non-negative meanings through constructing positive social representations. They endeavored to imagine their own identities and community in order to consolidate homosexuality as a concrete social way of being rather than just social practices. Trying to subvert constraints exerted on them by extant social inventions, gay men and lesbians in this study distanced themselves from non-positive social representations and voiced for themselves.

First, homosexuality was constructed as a social being instead of social practice that was associated with negative connotations. Homosexual(ity) in Chinese is a three-character word, namely *tongxinglian*. *Tong* means the same, *xing* means gender or sex, and *lian* refers to affection, attachment, attraction, and dating [27]. This word accentuates the sexual behavior of men who have sex with men in China more, and it carries shameful and negative connotations. As previous legal and medical documents had widely used it to refer to a certain kind of crime and mental disorder, the word itself represents more of an immoral and unnatural practice that is opposite to what the dominant discourses approve. Therefore, gay men and lesbians were more willing to refer themselves as *tongzhi* instead of *tongxinglian*. *Tongzhi* begins with the same character as *tongxinglian*, but the second character *zhi* means orientation, goal, and spirit. It is translated from “a Soviet communist term ‘comrade,’ which refers to the revolutionaries who shared a comradeship” [17]. Chinese in Republican China used the word initially, and then it was taken by both Communist and Nationalist Party to indicate people who struggled for communist or nationalist revolution [17]. The term *tongzhi* does not allude to same-sex sexual attractions nor sexual behaviors anymore, which obviates relating gay men and lesbians with their sexual practices [27]. Trying to insert themselves into the national narratives, participants preferred to use *tongzhi* to refer to themselves. The term not only disseminated a common-cause-pursuing spirit, but also separated the identity of a social being from social practice. The preference of using this word rather than *tongxinglian* was evident among the majority of respondents.

*As a tongzhi, our self-identification is very significant, and if we identify ourselves well with our tongzhi identity, then we will set our first step out toward our identity construction beautifully. (G2)*

G4 also used *tongzhi* to refer to his sexual identity when he was inquired about his identity realization.

*It was during my second year of university study when I realized that I was a tongzhi. (G4)*

Second, a community based on gay men and lesbians’ collective identity was constructed, operating as a collective representation to construct and circulate their own discourse as well as build up their cause. At the collective identity level, gay men and lesbians tended to include all same-sex oriented people they knew as *tongzhi*, as they wanted to both enlarge their community and propagandizing their cause of pursuing citizenship.

*Being members of the tongzhi community, we have to be responsible to ourselves and other members. First, we have to come out to our friends and family, trying to accumulate influence via snowball dissemination. We have to let the public know that homosexuality is not a disease nor being deviant, rather, it is an inborn thing. Additionally, as a community with human resources from various resources, why not think about fighting for our rights? Why not think about enjoying our happiness? Why not think about getting married in China? If I passed away, can I leave all my inheritance to my same-sex partner? These are all the questions we have to tackle if we want to be an updated version of tongzhi. (G2)*

The *tongzhi* community tried to set up a national boundary between themselves and those identities and practices they perceived as foreign. Though appropriating Western’s liberalism and queer theory into their own identity and community imagination, the *tongzhi* community distanced themselves from the West in terms of lifestyles, same-sex relationships, identity disclosure issues, STIs’ vulnerability, etc. [4]. These participants inserted the stereotypes from the general consciousness and dominant discourses perceiving Westerners to be promiscuous, superficial in relationship, as well as HIV-infected into their own discourse construing.

*The most frequent misunderstanding that I have encountered is the public’s stereotypical perceiving us as HIV-carriers. That is so untrue. We are unlike gay men in America or Europe who randomly pick anyone at a bar and conduct unprotected sex. We are controlled and constrained in terms of sex. In addition, the concept of using condoms has already been deeply rooted in the majority of tongzhi’s mind. (G10)*

G16 stated that unprotected and promiscuous sexual practices originated from Western countries, which were denied by the current *tongzhi* community.

*The popular ways for having a booty call all stem from foreign countries. The apps we use, the manners we adopt, and the venues where we pick up a friend with benefits all come from those countries. That is to say, some of the gays in China are influenced to be promiscuous and shallow, and it is not their true nature. NGOs within our community have embarked on educating gays to reject those negative impacts from Western cultures and learn to control and refrain themselves from superficial pleasures. More importantly, we strive to convey the idea that having a stable relationship will do them more good than have random sex to our gay fellows. (G16)*

Not only national boundaries were delineated by *tongzhi*, boundaries within the *tongzhi* community were also drawn between regions, genders, and gender performances. These efforts strove to situate the community into socially accepted qualities of civilized, educated, restrained, and diligent that were upheld by dominant discourses. First, according to participants, gay men and lesbians from rural areas were usually denied memberships in the *tongzhi* community, since many *tongzhi* from urban areas regarded them to be vulgar and less civilized, especially in light of sex behaviors.

*Gays from the countryside tend to have a more limited vision in terms of sex. They are more likely to deceive heterosexual women into marriage just for their own purpose, which is pathetic and selfish. This large group of gays have ruined our community’s reputation and intensified the public’s stigma on the whole tongzhi population. (G3)*

G17 conveyed the idea that many gay men from rural areas were less civilized, and they became the major source of STIs’ transmission.

*They are not so well informed of safe sex behaviors, especially wearing condoms during anal sex, so these people usually do not wear condoms during their intercourse with other men. Once they have opportunities to come to big cities, they immerse themselves in the unprotected and random sex, to which they have limited chance to access previously these people transmit many STIs, particularly HIV. We cannot say these rural gays part of our tongzhi community, as they are not civilized, refrained, and well informed as we are. (G17)*

Except for the regional boundary within the *tongzhi* community, gender boundary was also delineated within it between gays and lesbians based on the findings. Both parties refused to be associated with each other to some extent, as gays considered lesbians to be passive and indifferent, whereas lesbians perceived gays to be promiscuous and unreliable. They each wanted to retain their good social representations by discriminating the other’s undesirable representations.

*I think lesbians are persnickety, and they are very scheming. (G6)*

G2 mentioned that lesbians did not contribute much to the development of the *tongzhi* community.

*Lesbians take a very passive role when it comes to online or offline activities. They regard themselves to be part of our community, but they do nothing when this community needs them to speak out for it. Sometimes I am so angry when few lesbians volunteer to participate in our services and activities, because so many lesbians in our WeChat groups, but they just ignore the messages calling for their contributions. They dedicate little while sharing all the benefits and fruits we have fought, which is so unfair. (G2)*

Lesbian participants maintained negative attitude toward gays as well. L6 stated that lesbians were entirely different from gays in terms of their lifestyles.

*I do not reject marriage. Why would I reject it? I am not a gay, and only gays reject a stable relationship. Gays’ life is so messy and promiscuous. They do not pursue stable and long-lasting relationships, and they cannot reach them even if they desire. Because they are overall men, who think with the lower part of their bodies. Two men means two lower-part-body animals. That is why so many gays are HIV-positive, because they have no self-respect, so they can blame no one for their miserable life. (L6)*

L2 stated that gays were not able to sustain long-lasting relationships, which were different from lesbians.

*We are more reliable and stable when it comes to romantic relationships. Lesbians seldomly cheat on each other, because we retain higher sense of moral values than gays. Gays are totally opposite to us. They like seeking fun and pleasure, and good looking and masculine gays easily seduce them. More importantly, they have a low sense of morality, which explains that they could do anything for fun, money, and excitement. (L2)*

Based on the data, the last boundary set within the *tongzhi* community was on gender performances. Both gay men and lesbians upheld the idea that *tongzhi* should pay attention to their appearances, and they should learn to groom, work out, and dress with good taste. This was a sign that they were proud of who they were, and this will leave the public an impression that *tongzhi* were no worse and even better than the straight people were. *Tongzhi* who were fashionable, well groomed, and good-looking counted as popular, civilized, and positive within the *tongzhi* community. Additionally, both gay men and lesbians discriminated sissy gays, so gays who performed femininely were not popular within this community when it came to intimate relationships.

### 5.2. Pursuing Civilized Same-Sex Relationship

Whether coming out to their parents or not, some participants insisted on pursuing a same-sex relationship and did not surrender to the marital pressure. They strove hard to maintain their same-sex relationship civilized by keeping high sense of morality and being sincere to their intimate partners. This was due to their desire to decrease the social stigma imposed upon them and demonstrate to their parents that they could maintain a stable, caring, and long-lasting relationship that received little stigma even though their intimate partners were not heterosexual.

G19 now lived with his same-sex partner, and he had already come out to his mom. Though his mom accepted his gay identity, she refused to accept his way of living, as she still wanted him to marry a woman and live a life that the society considered as normal. Therefore, G19 had changed a lot to persuade his mom that his same-sex relationship was as moral and sincere as the relationship between heterosexual couples.

*Maintaining a stable same-sex relationship is quite hard, especially for gays, as there are so many distractions in our daily life that make it easy for us to betray our partners. That is what my mom is worried, because she thinks that we are not serious about our relationship. In order to assure my mom, my partner and I have changed our habits a lot. We do not go to nightclubs and bars very often and we have both found better jobs to maintain a decent living.*

According to participants, a stable and decent job will enable a longer lasting relationship for gay men and lesbians, which not only provide them higher social status and shield social stigma, but also decrease their conflicts regarding living expenditure if they cohabit with their partners. Moreover, participants believed that a decent job with high social status and good payments will prevent gay men from having infidelity affairs, as they value their reputation and thus would try to stay sincere to their partners just like heterosexual couples would do. Lesbian participants mentioned that they tend to show less interest in night clubs and random sex, and they tend to have a stabler relationship with their partners compared to their gay counterparts. Many lesbian participants maintain that it is important for them to maintain good habits to show the society that they are modern, independent, and highly educated people with good *suzhi* that are worth the public’s respect and equal treatment. They not only try to obtain higher education to find good jobs, but also pay serious attention to their appearance. They would rent a nice apartment with their partners and decorate it with taste, and they work out with their partners to stay fit, and they are active on their social network accounts to show their high-taste life to the public.

Another important symbol for gay participants to show their high *suzhi* is having protected sex by using condoms. Unprotected random sex would lead to the high risk of contracting STIs such as HIV, particularly among men who have sex with men. Chinese gay men are stigmatized severely because of this, and this becomes a major reason that the public often assigns negative connotation to gay men [28]. A new discourse was constructed among gay participants to show their high civilization and requirement of respectful treatment is the use of condoms for sexual behaviors. They regard using condoms as a critical way to protect themselves and their partners as well as their high sense of morality. By using condoms, they show their responsibility to themselves and their partners, also, they reduce the risk of becoming a bridge of transmitting STIs to the public, which demonstrates their responsibility to society. G18, aged 24, stated that one of the biggest misconceptions that the public have towards gay is that they are all AIDS carriers and thus they should isolate themselves from the public.

*Because of the insult and disrespect we have encountered so far, we have become ever more conscious about the safe sex issue. My friends and I will conduct the HIV test regularly every month, and we all reject random and unprotected sex. What is more, we always pay attention to the venue and place where we have sex, as we will not choose to go to places with dirty environment. (G18)*

Using condoms and having safe sex is also a critical sign to these participants, indicating that they are not hollow and shallow people who only indulge in sexual pleasure. In order to maintain a long-lasting relationship, many gay participants attach importance to the use of condoms, as both health and stable relationship are symbols of high *suzhi*.

### 5.3. Constructing a Flexible Life

Except for participants who pursed their same-sex identity and intimate life, some participants constructed a flexible life, for the sake of compromising their same-sex identity with the marital pressure. When faced with the marital pressure, these participants did not necessarily come out to their parents, meanwhile, they tried to strike a balance between making their parents satisfied and making their same-sex needs met.

Nominal marriage, a marriage formed by a gay and lesbian for the purpose of reproduction, shielding social stigma, and satisfying parents, is a choice that is frequently made by these people. Some participants dared not to disclose their identity to their parents out of the concern of being rejected by their parents or that this would deteriorate their parents’ health. However, marrying a heterosexual partner was not an option as well, as this would make them depressed and painful. As a result, many participants considered forming or have already formed a nominal marriage. They attached great positive connotation to nominal marriage by associating it with high sense of morality, as they were willing to sacrifice their own happiness to show their filial piety to their parents. L9 said:

*I cannot come out to my parents definitely, or else they would commit suicide because they are ashamed of me. They value their face more than my happiness. Finding a gay partner and marrying him would be the most suitable strategy for me, as I could enjoy my personal life and comfort my parents at the same time. (L9)*

Another gay respondent, G5 (aged 31), who got nominally married, stated that this type of marriage helped him to not only meet his parents’ expectations, but also enabled him to keep a same-sex identity. They maintained an open relationship in their daily life, and they accompanied each other to visit one’s parents when the need arose. As G5 mentioned:

*They (his parents) think that my marriage is a way to show my filial piety to them, because that is what they did when they were at my age. However, I can never tell them that always abiding by parents’ opinions and doing whatever they expect is not true filial piety. All I could do is to make them satisfied considering their age and health status, and meanwhile try to instill them with the modern culture little by little. Once they realize how unhappy I am by sacrificing my happiness to their expectations and face, they will feel sorry for that, as all parents do love their children deep in their heart. (G5)*

For participants who had same-sex partners, they were more likely to get nominally married, and the major purpose was to find a camouflage for their same-sex intimacy. On one hand, the same-sex identity was something they needed to hide from the public, thus finding a cover would protect them from social stigma and pressure while maintaining their relationship. On the other hand, marrying a heterosexual and walking into a heterosexual marriage would possibly have a negative influence on the same-sex relationship as the two-timing would not only consume their attention and affection for each other, but also expose their relationship and thus possibly ruin the relationship. Therein, nominal marriage was the best cover for them to both buffer against social pressure and stigma and maintain their relationship. G9, aged 29, was in a very stable same-sex relationship with his boyfriend for more than four years, and they both looked for prospective lesbian partners at the current stage. His boyfriend was now dating a lesbian and they had visited both sides’ parents as each other’s future spouse.

*My boyfriend and I live in the same city, and we have a common plan for our future. We plan to keep our relationship as we feel each other to be “the one”, and it is not easy to give up our relationship. We have discussed for a long time, and since my boyfriend is a little bit self-loathing because of his gay identity and his conservative environment, so it is not possible for him to come out. He cannot bear society’s discrimination nor losing his high-paid job, and hence a nominal marriage that could cover for him is the best strategy he can think of. I think, it would be better if we are both in a nominal marriage, not only because this provides us a nice cover, but also it makes the relationship more balanced as I will have my own fake family to tend to and not feel ignored during festivals when he needs to accompany his nominal wife. For our relationship, I am willing to cooperate with him and protect him from getting same-sex identity exposed. (G9)*

Moreover, nominal marriage could show gay men and lesbians’ high *suzhi* by demonstrating their sincerity and resourcefulness. On one hand, they did not need to deceive a straight person into marriage, as *tongqi* (straight women who marry gay men) were a group of people who were frequently mistreated and even abused by their gay husbands, and therefore, the public defies deceptive marriage. On the other hand, for gay men and lesbians who tended to get nominally married, they had to be more resourceful in terms of finding an appropriate nominal partner and being able to afford the prospective expense of wedding and festival family visiting gifts later on.

This kind of nominal marriage exposed some problems, though useful. The majority of the participants claimed that this kind of marriage resembled a cycling of lies, which meant that they had always to use one lie covering another. More severely, this would bring more pressure than being single, since the incoordination between partners and more requirements from parents could all take a toll on the people involved. It should be noticed that compared with gay men, lesbians considered the nominal marriage more like a show and they showed less willingness and determination to maintain it.

## 6. Discussion

The above narratives illustrate how Chinese gay men and lesbians try to use *suzhi* discourse to align themselves with the mainstream social values so as to obtain certain citizenship when faced with the marital pressure. Discourses in various domains associate homosexuality with stigmatizing and negative connotations, which promote the heteronormative ideology that deprives gay men’s and lesbians’ rights and benefits. The institutional power often associates gay men’s and lesbians’ same-sex identity and marital status with their job, and the disclosure of a same-sex identity might lead to dismissal. It works as a form of structural violence that governs, regulates, and controls individuals’ behavior [29]. The legal power is another form of power exercised by the marriage institution, as the legal discourse in China renders no protection for gay men and lesbians, and the only way this group of people want to obtain certain rights is pretending to be heterosexual. The last form of power is medical power, which brings forward covert and overt prejudice against gay men and lesbians. Gay men are still widely associated with HIV infection and transmission, and one study documents that even healthcare professionals hold this misconception, and their fear or homophobia poses a barrier to the health or well-being to GLB members, especially aging ones [30].

However, different from previous research that often put gay men and lesbians in an involuntary position in terms of power struggle (e.g., [31]), we find that some gay men and lesbians proactively deal with the marital pressure by associating themselves with high *suzhi*. They have established their own identity of high responsibility, well-educated and sincere, who deserve to be treated with equal respect to their straight counterparts. When faced with the marital pressure, they assert a same-sex identity strongly or seek nominal marriage for convenience, which echoes previous research [3,16]. However, different from existent findings, this paper perceives how Chinese gay men and lesbians’ deal with their marital pressure from a new angle of *suzhi* by identifying how they establish a high-*suzhi* identity and community as well as use *suzhi* to justify their different choices.

The heteronormative discourse carried out by Chinese marriage institution brought with it power inequality and manipulations, which aimed to maneuver gay men and lesbians into conformation with heteronormativity. Endeavoring to situate their discourse on dominant voices, this discourse made it difficult for gay men and lesbians to deny it. Jones [4] claims that China is now in the middle of a discursive revolution. Voices from various social aspects have now arisen competing for dominance in a more liberalized social atmosphere [4]. According to Gu [32], China used to be dominated by two major discourses after Mao’s death, and they are the revolution discourse and reform discourse. The former one embodies class struggle and enforces conformity, collectivism, and self-denial. The latter one stresses economizing and encourages individualism more (as Cited in [4] pp. 89–90). Contingent on the recent discourse, gay men and lesbians inserted the pursuit of intimate citizenship into their discourse in order to echo what dominant voices promulgate. This strategic maneuvering of voices from distinct discourses in order to distort, invert, or alter constraints in these discourses is a kind of power manipulation [4]. This is a strategy used in Aikido, where a less powerful party confronts a more powerful party, he or she should ride on the other’s power and ride it toward his or her own advantage [33]. This kind of tactic echoes with the Daoist philosophy of govern by doing nothing that goes against nature or accomplish a great task with little effort by clever maneuvers.

## 7. Conclusions

This is one of the few studies that took a leading step to study Chinese gay men and lesbians through a new perspective. By using qualitative interview, this study was able to explore Chinese gay men and lesbians’ different way of handling the pressure to marry through the *suzhi* lens. In order to align themselves with the mainstream social values so to make themselves more acceptable to Chinese society, participants took three approaches—constructing a positive same-sex identity and civilized community, pursuing civilized same-sex relationship, and constructing a flexible life. This study has the following implications. First, it concentrated on a special group of people in China whose voice are frequently weak and whose social well-being is precarious. Results generated by this study contribute to more and deeper understanding of Chinese gay men and lesbians. Besides, this study sheds light on how to understand gay men and lesbians’ justification of their intimate citizenship through the lens of *suzhi* discourse. Meanwhile, heteronormative discourse is a form of ideology that regulates and governs bodies, and it structures people’s everyday lives. It imposes assumptions about gender and sexuality on individuals—disciplining how sexual lives can be and what counts as an appropriate sexual life. The discourse is embedded in practices which are often invisible. To subvert this discourse, people, particularly service providers such as social workers, must critically interrogate how their practice—though unwillingly—reinforces and maintains heteronormativity. Moreover, service providers must also carefully examine how heteronormativity is embedded in programs that marginalize sexual minorities continuously. Future research that uses quantitative methods to further testify the results of this paper will be needed to better understand the moderators and mediators of these approaches.

## Data Availability

Not applicable.
